# Influence of envelope waveform on ITD sensitivity of neurons in the auditory midbrain

**DOI:** 10.1152/jn.01048.2015

**Published:** 2017-07-12

**Authors:** David Greenberg, Jessica J. M. Monaghan, Mathias Dietz, Torsten Marquardt, David McAlpine

**Affiliations:** ^1^UCL Ear Institute, London, United Kingdom;; ^2^Department of Linguistics, Australian Hearing Hub, Macquarie University, Sydney, New South Wales, Australia; and; ^3^Medizinische Physik and Cluster of Excellence Hearing4all, Universität Oldenburg, Oldenburg, Germany

**Keywords:** envelope ITD, ITD-sensitive neurons, amplitude modulated

## Abstract

Interaural time differences (ITDs) conveyed by the modulated envelopes of high-frequency sounds can serve as a cue for localizing a sound source. Klein-Hennig et al. (*J Acoust Soc Am* 129: 3856, 2011) demonstrated the envelope attack (the rate at which stimulus energy in the envelope increases) and the duration of the pause (the interval between successive envelope pulses) as important factors affecting sensitivity to envelope ITDs in human listeners. Modulated sounds with rapid attacks and long pauses produce the lowest ITD discrimination thresholds. The duration of the envelope’s sustained component (sustain) and the rate at which stimulus energy falls at the offset of the envelope (decay) are only minor factors. We assessed the responses of 71 single neurons, recorded from the midbrains of 15 urethane-anesthetized tri-colored guinea pigs, to envelope shapes in which the four envelope components, i.e., attack, sustain, decay, and pause, were systematically varied. We confirmed the importance of the attack and pause components in generating ITD-sensitive responses. Analysis of neural firing rates demonstrated more neurons (49/71) show ITD sensitivity in response to “damped” stimuli (fast attack and slow decay) compared with “ramped” stimuli (slow attack and fast decay) (14/71). Furthermore, the lowest threshold for the damped stimulus (91 μs) was lower by a factor of 4 than that for the temporally reversed ramped envelope shape (407 μs). The data confirm the importance of fast attacks and optimal pause durations in generating sensitivity to ITDs conveyed in the modulated envelopes of high-frequency sounds and are incompatible with models of ITD processing based on the integration of sound energy over time.

**NEW & NOTEWORTHY** Using single-neuron electrophysiology, we show that the precise shape of a sound’s “energy envelope” is a critical factor in determining how well midbrain neurons are able to convey information about auditory spatial cues. Consistent with human behavioral performance, sounds with rapidly rising energy and relatively long intervals between energy bursts are best at conveying spatial information. The data suggest specific sound energy patterns that might best be applied to hearing devices to aid spatial listening.

## Abstract (precis)

Using single-neuron electrophysiology, we show that the precise shape of a sound’s “energy envelope” is a critical factor in determining how well midbrain neurons are able to convey information about auditory spatial cues. Consistent with human behavioral performance, sounds with rapidly rising energy and relatively long intervals between energy bursts are best at conveying spatial information. The data suggest specific sound energy patterns that might best be applied to hearing devices to aid spatial listening.

the ability to determine the location of a sound source on the horizontal plane relies on detecting differences in the timing and intensity of the sound at the two ears. The classic duplex theory of binaural hearing (Strutt 1907) posits that humans exploit interaural time differences (ITDs) predominantly for localizing low-frequency (<1,500 Hz) sound sources by virtue of sensitivity to the ongoing temporal fine structure at each ear. Sources emitting frequencies higher than this are localized by virtue of the “acoustic shadow” generated at the ear farther from the source. The resulting interaural level differences (ILDs) are larger for sources located more laterally and increase with increasing frequency. Despite its elegance, the dichotomy suggested by the duplex theory is not strict. In particular, listeners are also able to exploit ITDs conveyed in the modulated envelopes of high-frequency sounds to perform spatial listening tasks (Bernstein and Trahiotis 1994, 2002; Henning 1974; Klumpp and Eady 1956; McFadden and Pasanen 1976; Nuetzel and Hafter 1976). In cases of both low- and high-frequency sounds, physiological sensitivity to ITDs relies on the generation of action potentials at the synapse of the inner hair cells, phase-locked to either the stimulus fine structure for low-frequency sounds or to the stimulus envelope for high-frequency sounds. Action potentials propagating through the auditory pathway converge onto neurons in the two principal nuclei of the superior olivary complex (SOC), the medial and lateral superior olives (MSO and LSO) (Batra et al. 1997; Caird and Klinke 1983; Goldberg and Brown 1968), where a process of binaural cross-correlation occurs (Yin and Chan 1990), either of action potentials phased-locked to the temporal fine structure (for frequencies below 1–1.5 kHz) or to the stimulus envelope (above ~2 kHz; Joris et al. 2004; Palmer and Russell 1986). The SOC transmits this encoded ITD information to the inferior colliculus (IC), the major nucleus of the auditory midbrain where neural sensitivity to ITDs, including sensitivity to envelope ITDs conveyed, for example, by sinusoidally amplitude-modulated (SAM) tones, is widely reported (Batra et al. 1989, 1993; Griffin et al. 2005; Yin et al. 1984).

In seeking to establish the extent to which binaural processing of low- and high-frequency ITD cues is similar, Bernstein and Trahiotis (2002) assessed human sensitivity to high-frequency envelope ITDs using so-called “transposed tones,” high-frequency tones modulated in such a way that the presumed temporal pattern of neural output resembles that of low-frequency pure tones (Colburn and Esquissaud 1976). In addition to observing ITD thresholds to be lower than for the more commonly employed SAM tones, they also reported ITD thresholds for transposed tones to be as good as or better (in the case of modulation rates below 128 Hz) than those obtained for low-frequency pure tones. The same authors (Bernstein and Trahiotis 2009) later generated stimuli with envelope parameters intermediate to those of SAM and transposed tones by manipulating independently the pause duration and the attack and decay steepness of the waveform envelope. They found that a graded increase in pause duration and a reduction in the steepness of the attack and decay phases of the envelope resulted in a graded improvement in ITD thresholds. This accords with physiological evidence demonstrating enhanced neural sensitivity to ITDs with envelopes that incorporate extended sections of zero amplitude (such as transposed tones) compared with those without (such as SAM tones) (Dreyer and Delgutte 2006; Griffin et al. 2005).

The influence of envelope shape on ITD sensitivity in human listeners was further analyzed by Klein-Hennig et al. (2011), who manipulated independently the duration of the four distinct envelope segments: attack, sustain, decay, and pause (see Fig. 1*A*) of modulated high-frequency tones. In general, envelope shapes with a short attack duration and a long pause generated the lowest ITD thresholds, whereas sustain and decay segments had little influence on performance. Most critically, temporally asymmetric envelope shapes with a short attack segment and a long decay (referred to as “damped”) elicited considerably greater ITD sensitivity in human listeners than spectrally identical, but temporally reversed, “ramped” stimuli. Many existing models of binaural interaction cannot explain this difference, because their performance often depends on the spectrum of the preprocessed signal (Bernstein and Trahiotis 1996; Dietz et al. 2012), which is identical in the case of the time-reversed ramped and damped stimuli (see Fig. 1*C*).

**Fig. 1.**
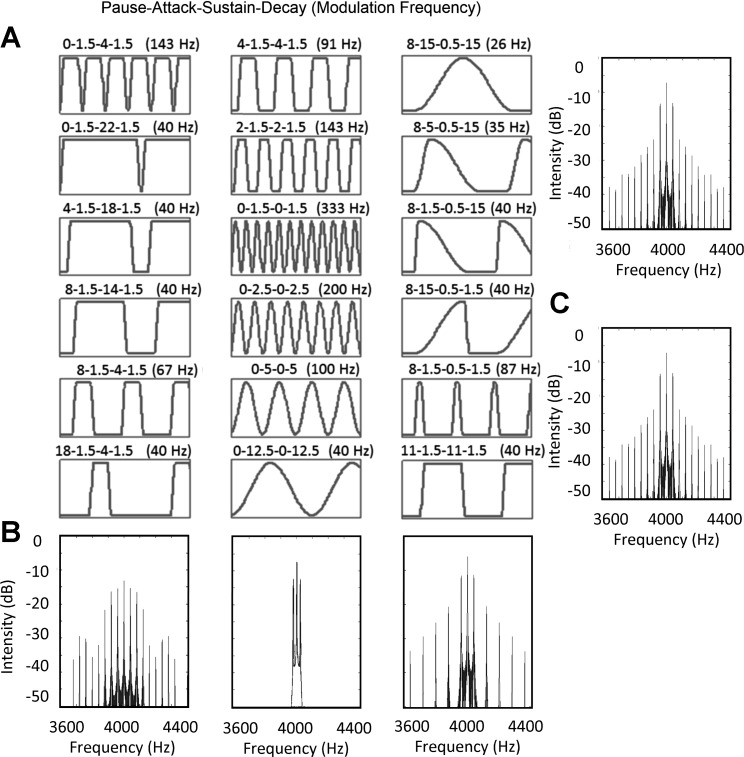
*A*: illustrations of the 18 envelope shapes used to assess the ITD sensitivity of the midbrain neurons. Above each schematic is the duration (in ms) of each pause, attack, sustain, and decay segment (and corresponding modulation frequency). The envelope shapes are ordered according to response comparisons described in results. *B*: power spectra of the 3 envelope shape modulations in *bottom* row of *A*. *C*: power spectra of the 2 temporally asymmetric envelope shapes, damped and ramped, are observed to be identical.

In this report, we investigate the envelope ITD sensitivity of IC neurons by manipulating independently the duration of the attack, sustain, decay, and pause segments of the envelope in a manner similar to that employed by Klein-Hennig et al. (2011). We demonstrate that the responses of ITD-sensitive neurons are similarly sensitive as human psychoacoustic performance to changes in attack and pause duration. The data indicate the importance of envelope shape over factors such as modulation rate: sounds with the same modulation rate, but different envelope shapes, can generate considerably different ITD sensitivity, to the extent that they are ITD insensitive to one envelope shape and highly sensitive to another. The clear importance of the envelope shape, in particular the sharp attack phase required to generate significant neural tuning for ITD, has implications for interventions such as bilateral cochlear implantation (Monaghan and Seeber 2016), where ITD information is available only in the modulated envelope of sounds and not in the temporal fine structure.

## METHODS

### 

#### Experimental materials and methods.

A selection of the data presented in this article was published as part of a conference proceedings (Dietz et al. 2013a). The experimental methods used are identical to those reported previously. The experimental methods, including surgical procedures and recording equipment, are also described in detail in Dietz et al. (2014).

#### Animals, surgery, stimulation, and recording hardware.

Single-neuron recordings were made from 71 neurons in the IC of 15 adult tri-colored guinea pigs under urethane anesthesia (20% solution, 1.5 g/kg ip dosage) at the UCL Ear Institute. These experiments were carried out in accordance with the Animal (Scientific Procedures) Act of 1986 of Great Britain and Northern Ireland.

Rimadyl (50 mg/ml solution, 2-mg constant dosage, nonsteroidal anti-inflammatory drug and analgesic), buprenorphine (0.3 mg/ml solution, 0.05 mg/kg dosage, opioid), and Colvasone (2 mg/ml solution, 2 mg/kg dosage, corticosteroid with an anti-inflammatory action) were injected subcutaneously at the beginning of each experiment. A craniotomy was performed to expose the cerebral cortex overlying the IC, and the covering dura was removed. The bullae were vented by insertion of cannulas and were sealed with Vaseline to equalize middle ear and outside air pressures. A parylene-coated tungsten microelectrode (1–2 MΩ; World Precision Instruments, Sarasota, FL) was positioned above the IC and advanced ventrally using a motorized micromanipulator (model SM-5, Luigs and Neumann, Ratingen, Germany). At the end of each experiment, animals were administered a lethal dose of pentobarbital sodium (200 mg/ml ip).

Sounds were produced using Tucker Davis Technologies (TDT; Alachua, FL) digital signal processing hardware. TDT’s Brainware software controlled the sequence and parameters of the stimuli that were computed on an RP2.1 processor (TDT) using the Real Time Processor Visual Design Studio (RPvdsEx). Stimuli were generated with a 48.828-kHz sampling rate and scaled such that their peak voltages were 5 V at the digital-to-analog converter outputs. These signals were attenuated to achieve the desired level for the experiments by using PA5 (system III) modules (TDT). Sounds were delivered by Etymotic ER-4S headphones with the common reference wire separated to abolish cross talk. Microphones (FG3452; Knowles Acoustics, Burgess Hill, UK) were used to measure the stimulus within a few millimeters of the tympanic membrane to ensure that the sounds delivered to each ear were well matched.

A binaural search stimulus consisting of ongoing 50-ms presentations of diotic pure tones was used to isolate neurons by eliciting measurable electrical activity. A first approximation of a neuron’s response threshold and characteristic frequency (CF) were made, and the threshold and CF were then confirmed by recording a frequency-vs.-level response, followed by a test of ITD sensitivity. The confirmed CF of the neuron was used as the carrier frequency for the envelope modulation. If the CF was sufficiently high to preclude ITD sensitivity being conveyed in the signal fine structure (above 1.4 kHz), as well as the neuron being readily responsive to ongoing stimulation and sensitive to changes in ITD, the full envelope shape stimulation protocol was presented. A trigger point was set above the noise floor determined by the experimenter. Spike clusters were selected on the basis of spike positive and negative spike magnitude deflections. Because of the large amount of data being recorded and stored, it was not possible to also record raw traces.

Each cycle of the stimulus envelope was constructed from four segments, identical to those used by Klein-Hennig et al. (2011): *1*) a pause segment with zero amplitude, *2*) an attack segment identical to the rising portion of a squared sinusoid, *3*) a sustain segment with a maximum amplitude, and *4*) a decay segment identical to the falling portion of a squared sinusoid. The envelope shape parameters are presented in Table 1 and are illustrated in Fig. 1. Stimuli were constructed with a carrier frequency equal to the neuronal CF (as determined audiovisually) and were 100% modulated. The ITD was applied after the carrier was multiplied by the envelope, resulting in a full waveform shift. The “fine” recording range was between −2 and +2 ms and consisted of responses to 25 evenly spaced ITDs, i.e., a 167-µs spacing. The “coarse” recording range extended over a maximum set of ITDs between −8.33 and +28.33 ms, and a 1.67-ms ITD spacing was used.

**Table 1.** Parameters of the envelope modulation stimuli used in experiments

Condition Number	Pause, ms	Attack, ms	Sustain, ms	Decay, ms	Period Duration, ms	Modulation Frequency, Hz	Condition Name
*1*	0 (0)	12.5 (10)	0 (0)	12.5 (10)	25	40 (50)	40-Hz SAM
*2*	11(13.1)	1.5 (1.25)	11 (13.1)	1.5 (1.25)	25	40 (35)	40-Hz PSW
*3*	0 (0)	1.5 (1.25)	22 (17.5)	1.5 (1.25)	25	40 (50)	0-ms Pause PSW
*4*	4 (4.4)	1.5 (1.25)	18 (13.1)	1.5 (1.25)	25	40 (50)	4-ms Pause PSW
*5*	8 (8.8)	1.5 (1.25)	14 (8.8)	1.5 (1.25)	25	40 (50)	8-ms Pause PSW
*6*	18 (17.5)	1.5 (1.25)	4 (0)	1.5 (1.25)	25	40 (50)	18-ms Pause PSW
*7*	8 (10)	1.5 (1.25)	0.5 (0)	15 (18.75)	25	40 (33)	40-Hz Damped
*8*	8 (10)	15 (18.75)	0.5 (0)	1.5 (1.25)	25	40 (33)	40-Hz Ramped
*9*	0	1.5 (1.25)	0	1.5	3	333	333-Hz SAM
*10*	0	2.5	0	2.5	5	200	200-Hz SAM
*11*	0 (0)	5 (5)	0 (0)	5 (5)	10	100 (100)	100-Hz SAM
*12*	2	1.5	2	1.5	7	143	143-Hz PSW(*i*)
*13*	4 (3.75)	1.5 (1.25)	4 (3.75)	1.5	11	91 (100)	91-Hz PSW
*14*	0	1.5	4	1.5	7	143	143-Hz PSW(*ii*)
*15*	8 (13.1)	1.5 (1.25)	4 (4.4)	1.5 (1.25)	15	67 (50)	67-Hz PSW
*16*	8 (13.1)	1.5 (1.25)	0.5 (0)	1.5 (1.25)	11.5	87 (64)	87-Hz PSW
*17*	8 (0)	15 (10)	0.5 (0)	15 (10)	38.5	26 (50)	8-ms Pause SAM
*18*	8	5	0.5	15	28.5	35	35-Hz Damped

The carrier frequency of a sinusoidally amplitude-modulated (SAM) tone is the characteristic frequency of the neuron to be assessed. The pseudo square-wave (PSW) tones differ from the SAM tones in having pause and sustain durations >0 ms. Numbers in parentheses are the values of the parameters in the most closely corresponding condition of Klein-Hennig et al. (2011).

The stimulus duration was always an integer multiple of the cycle duration (starting and stopping in the modulation trough) and as close to 1 s as possible (990–1,010 ms). The interstimulus interval was at least 300 ms. Responses to 18 different envelope shapes with an average of 38 different ITDs were recorded between 4 and 10 times (mean = 7.8), dependent on how well the isolation of the neuron was maintained. The stimulus presentation order was chosen so as to record conditions with identical envelope shapes en bloc. Within each block, the ITD was successively increased. Thereafter, for the next run, the presentation order was reversed. In these reversed runs the carrier was inverted in one channel to cancel out potential residual tuning to the carrier interaural phase difference (IPD; Joris 2003). Stimuli were presented with constant maximum amplitude ~20 dB above the pure-tone threshold at a neuron’s CF.

Because we are only interested in the response to ITDs in the ongoing stimulus (rather than the stimulus onsets), we excluded the first 100 ms of spike responses from the analysis. Similarly, the psychoacoustic studies of interest (e.g., Klein-Hennig et al. 2011) employed ramped onsets to reduce the effect of onset ITDs on the human performance.

A discrimination index is required to set the criteria from which to extrapolate the ITD just-noticeable difference (JND), the smallest change in ITD that will elicit a statistically significant change in neural response. The index D, the standard separation (Sakitt 1973), has the following properties that make it ideal as part of a discriminability index in the present context: *1*) it is independent of assumptions about the underlying response distributions, *2*) it is independent from the units used for rating task responses, *3*) it allows the comparison of different neurons from the same task and of the same neurons with different tasks, and *4*) it is affected by changes in variance of responses. D is calculated as follows:

$${\text{D}}_{\text{(ITD1,ITD2)}}=\frac{{\text{R}}_{\text{(ITD2)}}-{\text{R}}_{\text{(ITD1)}}}{\sqrt{{\text{SD}}_{\text{(ITD1)}}{\text{SD}}_{\text{(ITD2)}}}}$$

where R_(ITD2)_ and R_(ITD1)_ are the mean spike responses in response to two different envelope ITDs and SD_(ITD1)_ and SD_(ITD2)_ are the standard deviations of the respective response distributions. A random rating would give D = 0, and perfect discrimination would produce an infinite D. The neuron population characteristics are defined using the criterion of D = 1.

To calculate the ITD JND for a particular neuron and envelope shape, the ITD at which D = 1 is reached is calculated. The smallest difference between two ITDs, in response to the “fine” recordings, which reached the D criteria, was taken as the ITD JND. This point will occur between two test ITDs (for the fine-grained recordings, responses were recorded at 0.167-ms ITD intervals), and so linear interpolation is used to calculate the ITD JND.

Because the range of neural CFs extended down to the range associated with sensitivity to ITDs conveyed in the temporal fine structure of sounds (<2 kHz), we employed the “diff-corr” technique (Joris 2003) to assess phase locking to the carrier of all neurons with CF <2 kHz. In this technique, ITDs conveyed in amplitude-modulated sounds are compared for opposite polarities of the carrier phase. For neurons (or components of neural responses) sensitive to ITDs conveyed in the carrier, these two responses cancel each other, leaving an unmodulated response (or less modulated for neurons in which both carrier and envelope ITD sensitivity are evident). Because we sign-inverted the phase of the carrier on half of the presentations in all of our experiments, this enabled us to compare neural responses to the two polarities of the carrier phase, which we did for all six neurons with CF <2 kHz. Only the neuron with the lowest CF (1.4 kHz) showed any sensitivity to carrier ITD, with modulation of the neural response clearly related to the 1.4-kHz carrier frequency. No other neurons showed evidence of ITD sensitivity to the carrier.

## RESULTS

Responses to 18 envelope shapes were obtained from the right IC of 15 tri-colored guinea pigs. Recordings were made from 71 neurons. Post hoc analysis of each neuron included assessment of rate-vs.-ITD functions and ITD thresholds. CFs of all 71 neurons were ≥1.4 kHz (1.4–12.2 kHz, median = 4.3 kHz). The shortest stimulus-response latencies recorded from each neuron, in response to a stimulus with a short (1.5 ms) attack segment, ranged from 14 to 42 ms, in agreement with existing studies of first-spike latency of IC neurons (Tan et al. 2008; Zohar et al. 2011).

### 

#### Firing patterns evoked by envelope ITD stimuli with short or long attack and decay segments.

**Fig. 2.**
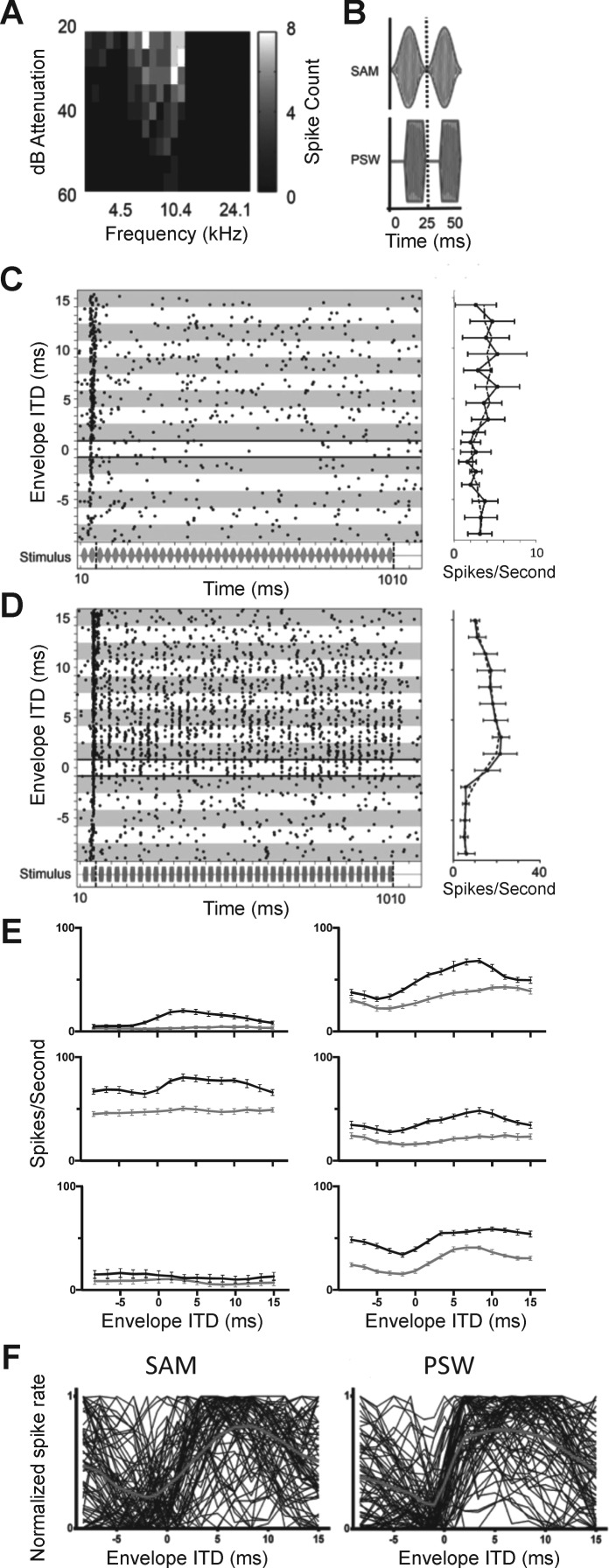
*A*: frequency vs. level response area. *B*: illustrations of the SAM and PSW envelope shapes assessed in Fig. 2. *C* and *D*: responses to envelope ITD stimuli for SAM (*C*) and PSW (*D*) modulation shapes in the same neuron show different ITD tuning. *Left*, spike raster displays for responses to SAM- and PSW-shaped modulations in 1 neuron (stimulus shown under spike raster). Each row of dots in the raster plots shows the neuron’s spike times in response to one presentation of a 10.4-kHz pure tone (the neuron’s CF) with a modulation rate of 40 Hz. The abscissa of each dot indicates the spike time with relation to stimulus onset. Envelope ITD was varied successively between −8.33 and 15 ms in 1.67-ms steps. Responses at each envelope ITD were recorded a total of 8 times and are presented together within the alternating gray and white rows. The bold horizontal lines highlight spikes times in response to zero envelope ITD. The vertical dashed line in the stimulus waveform plots indicates the 100-ms point after which the neural responses were taken to be in response to the ongoing stimulus and not the stimulus onset. Only responses to the ongoing stimulus were used to calculate ITD JNDs. *Right*, mean spike rates as a function of ITD (solid black lines) alongside the 3-point-average (a moving mean of 3 points) spike rate (dashed black line). Error bars are ±SD. *E*: 3-point-average spike rate as a function of ITD for SAM (gray lines)- and PSW (black lines)-shaped modulations measured in 6 different single neurons from 6 different ICs. *F*: normalized average spike rate as a function of ITD for SAM- and PSW-shaped modulations for all 71 neurons (1 trace per neuron). The bold gray line indicates the average normalized rate-ITD function for all 71 neurons.

The response of a typical IC neuron sensitive to envelope ITD is shown in Fig. 2, *A*–*D*. CF was judged to be 10.4 kHz, confirmed by the frequency-vs.-level response area (Fig. 2*A*). Raster plots (Fig. 2, *C* and *D*, *left*) show responses to CF tones modulated with SAM or pseudo square-wave (PSW) modulation shapes, and interaurally delayed over a range of ITDs between −8.33 and 15 ms in steps of 1.67 ms. Phase locking to the 40-Hz envelope modulation is evident to the PSW envelope shape (Fig. 2*D*) but not obviously so to the SAM (Fig. 2*C*). ITD tuning is present for the PSW envelope shape, indicated by higher discharge rates at favorable ITDs and reduced rates at unfavorable ITDs, compared with the SAM envelope shape, for which discharge rates remain low across all envelope ITDs assessed. Mean responses to SAM and PSW envelope shapes across all envelope ITDs are displayed as rate-vs.-ITD functions in Fig. 2, *C* and *D*, *right*.

Figure 2*E* compares rate-vs.-ITD functions for SAM and PSW modulation shapes for six IC neurons from six different animals. For most neurons, the rate-vs.-ITD functions have different shapes for the SAM and PSW stimuli, with SAM eliciting a lower overall spike rate and a less modulated discharge rate. Figure 2*F* plots, for all 71 neurons, the normalized firing rate as a function of envelope ITD for the SAM (Fig. 2*F*, *left*) and PSW (Fig. 2*F*, *right*) modulation shapes. Both stimuli elicit response functions in which firing rates are most steeply modulated around zero ITD, with PSW modulation eliciting a more steeply inclined change in discharge rate than SAM.

**Fig. 3.**
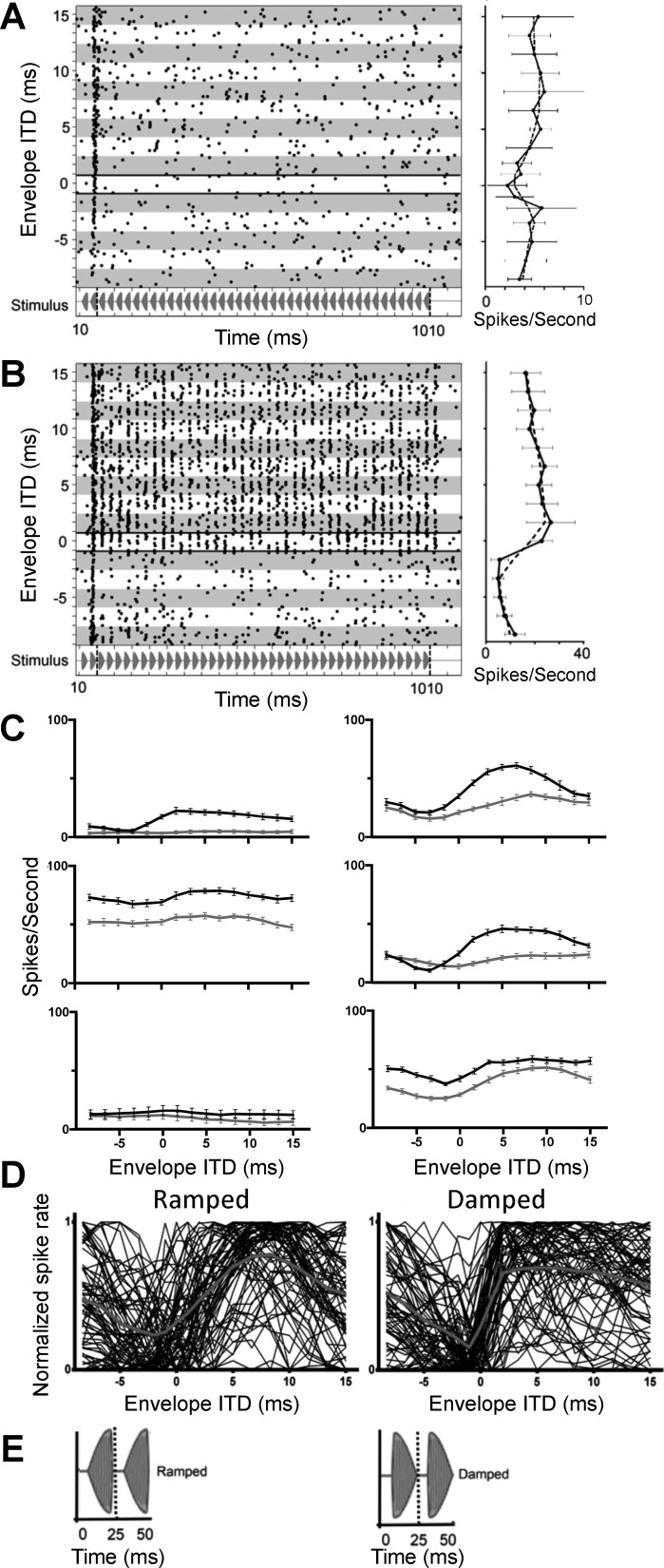
*A* and *B*: responses to envelope ITD stimuli for ramped and damped modulation shapes in the same neuron as in Fig. 2. Ramped-shaped modulations (*A*) produce ITD tuning equivalent to SAM tones (Fig. 2*C*), and damped-shaped modulations (*B*) produce ITD tuning equivalent to PSW-shaped modulations (Fig. 2*D*). *Left*, spike raster displays for responses to ramped and damped modulation shapes in 1 neuron (stimulus shown under spike raster). All other measurement parameters and annotations are the same as in Fig. 2. *Right*, mean spike rates as a function of ITD (solid black lines) alongside the 3-point-average spike rate (dashed black line). Error bars are ±SD. *C*: 3-point-average spike rate as a function of ITD for ramped (gray lines)- and damped (black lines)-shaped modulations measured in 6 different single neurons from 6 different ICs. *D*: normalized average spike rate as a function of ITD for ramped (*left*)- and damped (*right*)-shaped modulations for all 71 neurons (1 trace per neuron). The bold gray line indicates the average normalized rate-ITD function for all 71 neurons. *E*: illustrations of the ramped and damped envelope shapes assessed in Fig. 3.

Responses of the same neuron as in Fig. 2, *A*–*D*, are shown in Fig. 3, *A* and *B*, for the ramped and damped modulation shapes, respectively, again over the range of ITDs from −8.33 to 15 ms in steps of 1.67 ms. Phase locking to the modulation envelope is clearly evident for the damped stimulus (Fig. 3*B*), but not for the ramped stimulus (Fig. 3*A*). Recall that these two stimuli have identical spectra. ITD tuning is also clearly present for the damped stimulus, with discharge rates increasing at favorable ITDs and decreasing at unfavorable ITDs, compared with the ramped envelope shape, for which discharge rates remain low across all ITDs. Mean responses to ramped and damped envelope shapes across all envelope ITDs are shown as rate-vs.-ITD functions in Fig. 3, *A* and *B*, *right*.

Figure 3*C* compares spike rates as a function of envelope ITD for ramped (gray lines) and damped modulations (black lines) for the same six neurons as shown in Fig. 2*E*. For most neurons, the ramped modulation elicited a lower overall spike rate, and a smaller modulation of this rate, than did the damped modulation. Figure 3*D* plots the normalized firing rate as a function of envelope ITD for ramped (Fig. 3*D*, *left*) and damped modulations (Fig. 3*D*, *right*) for all 71 neurons. The damped stimulus elicits a steeper change in spike rate as a function of envelope ITD, especially around zero ITD, compared with the ramped stimulus.

#### Response patterns evoked by envelope ITD stimuli with short or long sustain and pause segments.

We also assessed the influence of sustain and pause segments on ITD sensitivity of IC neurons using PSW envelopes, i.e., envelopes with short (1.5-ms) attack and decay segments. The sustain and pause segments of the envelope were then varied in three different ways: *1*) varying the pause duration with a fixed sustain duration, *2*) varying sustain with fixed pause, and *3*) with a constant modulation cycle duration of 25 ms. Assessing responses with respect to these manipulations of the stimulus envelope will help determine whether differences in ITD sensitivity are due to the varying segment lengths per se or, rather, are the result of unavoidable covariations in other factors such as the modulation frequency.

**Fig. 4.**
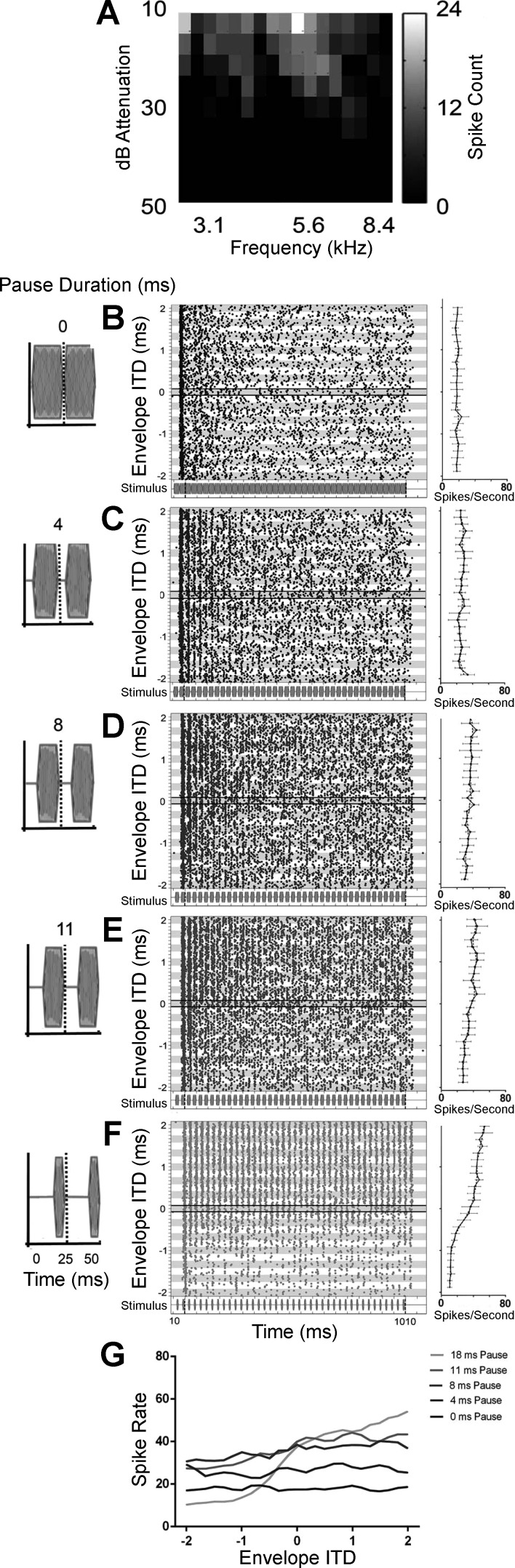
*A*: frequency-vs.-level response area. *B–F*: responses to envelope ITD stimuli (*middle*) for PSW-shaped modulations with pause duration segments of 0 (*B*), 4 (*C*), 8 (*D*), 11 (*E*), and 18 ms (*F*) in the same neuron show different ITD tuning. Spike raster displays responses to 5 different PSW-shaped modulations in 1 neuron (stimulus illustration shown under spike raster and magnified at *left*). Each row of dots in the raster plots shows the neuron’s spike times in response to one presentation of a 5.6-kHz pure tone (the neuron’s CF) with a modulation rate of 40 Hz. Envelope ITD was varied successively between −2 and 2 ms as the “fine” recording range consisting of responses to 25 evenly spaced ITDs, i.e., with 167-µs steps. Responses at each envelope ITD were recorded a total of 8 times and are presented together within the alternating gray and white rows. The bold horizontal lines highlight spikes times in response to zero envelope ITD. *Right*, mean spike rates as a function of ITD (solid black lines) alongside the 3-point-average spike rate (dashed black line). Error bars are ±SD. *G*: 3-point-average spike rates as a function of ITD for the 5 PSW-shaped modulations measured in *B–F* for comparison.

Responses of a second, typical IC neuron (CF = 5.6 kHz) sensitive to envelope ITDs are shown in Fig. 4. Raster plots (Fig. 4, *B*–*F*) show responses to PSW envelope shapes that were interaurally delayed over a range of ITDs between −2 and +2 ms in 0.167-ms, steps. In Fig. 4, *B*–*F*, the pause duration is increased from 0 to 18 ms and the sustain duration decreased from 22 to 4 ms, equivalent to a duty cycle that decreases from 25 to 7 ms while maintaining a constant modulation frequency of 40 Hz (corresponding stimuli illustrated on the left). Phase locking to the envelope of the stimulus appears greatest for the PSW envelope with the longest pause and shortest sustain durations (Fig. 4*F*). Rate-vs.-ITD functions to each envelope shape are plotted in Fig. 4, *C*–*G*, *right*. ITD tuning also appears greatest for the PSW envelope shape with the longest pause and shortest sustain durations (Fig. 4*F*). Discharge rates are lowest for the PSW envelope shape with the shortest pause duration and longest sustain duration (Fig. 4*B*). Figure 4*G* compares rate-vs.-ITD functions for the same the envelope shapes as in Fig. 4, *B*–*F*. For this (typical) neuron, the response rate and ITD tuning increase as the duty cycle is reduced.

**Fig. 5.**
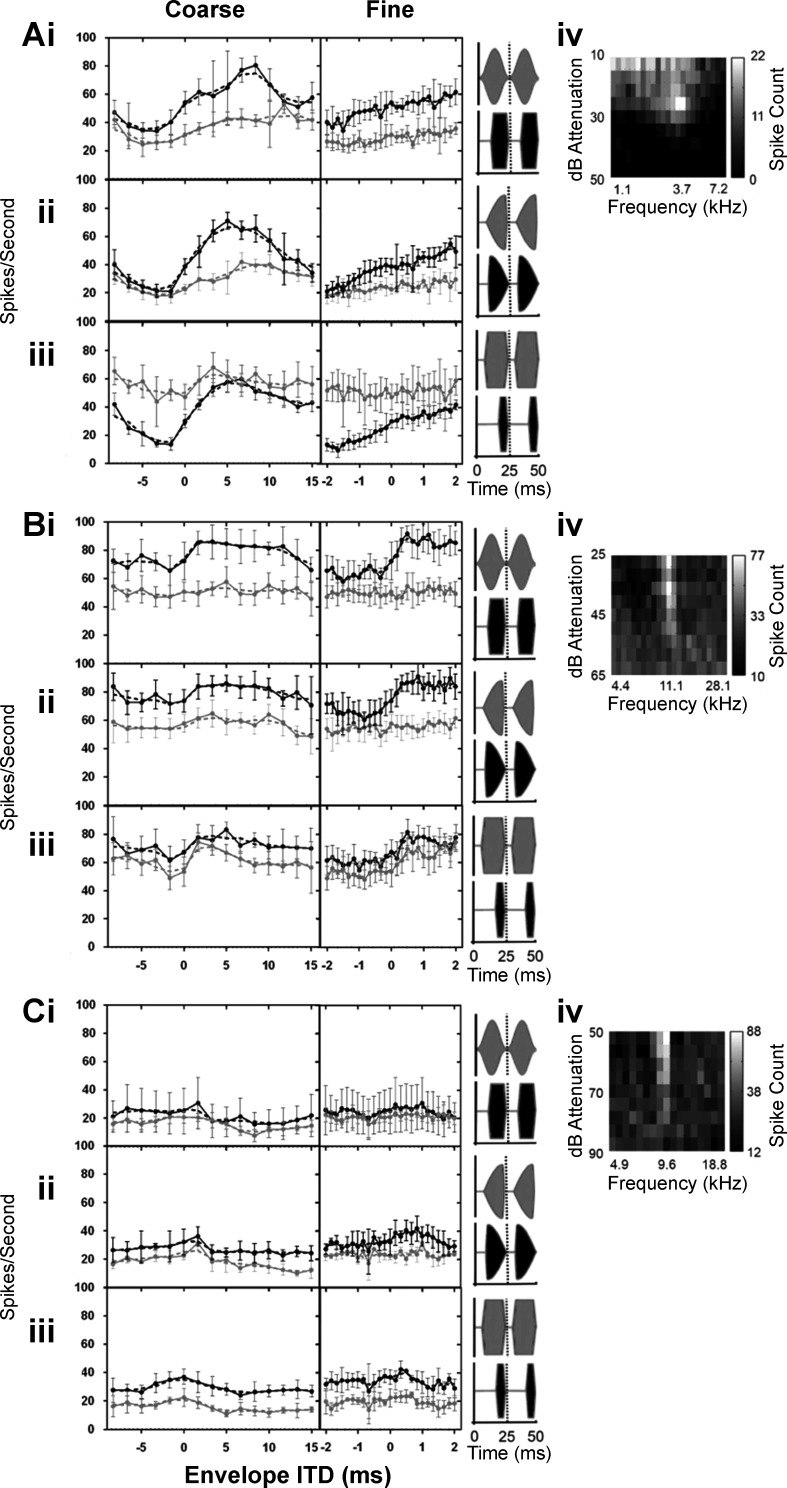
Rate-vs.-ITD functions for 3 IC neurons (*A–C*) for 3 key envelope shape comparisons: PSW vs. SAM (*i*), damped vs. ramped (*ii*), and short duty cycle vs. long duty cycle (*iii*). The coarse (*left*) and fine recordings (*right*) are shown. *Aiv–Civ*: frequency-vs.-level response areas.

Figure 5 plots rate-vs.-ITD functions for a further three IC neurons (*A–C*) and for three key envelope shape comparisons; PSW vs. SAM (*i*), damped vs. ramped (*ii*), and short duty cycle vs. long duty cycle (*iii*). Two ITD ranges were used for the recordings, a coarse range from −8.33 to +15 ms in 1.67-ms steps (Fig. 5, *A–C*, *left*) and a fine-grained range from −2 to +2 ms in 0.167-ms steps (Fig. 5, *A–C*, *right*). The neurons represented in both Fig. 5*A* and Fig. 5*B* are highly sensitive to envelope ITDs, whereas that in Fig. 5*C* is less so. In all instances, when a neuron is sensitive to envelope ITDs, the envelope shape with the shorter attack segment elicits the greater spike rate as well as the greater change in spike rate as a function of ITD. When the attack segment is of equal duration (as in Fig. 5, *Aiii–Ciii*) and the influence of the pause and sustain segment durations are assessed, the greatest changes in spike rate occur when then the pause segment is long, whereas the duration of the sustain segment appears to have comparatively little influence on either the overall spike rate or the modulation of the spike rate as a function of ITD.

**Fig. 6.**
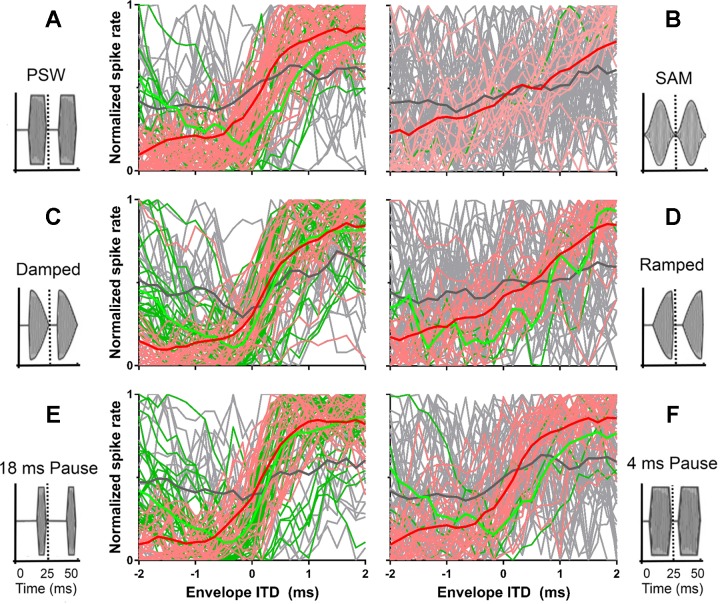
*A–F*: normalized rate-ITD functions for all 71 neurons for PSW (*A*), SAM (*B*), damped (*C*), ramped (*D*), short duty cycle (*E*) and long duty cycle (*F*). All normalized rate-ITD functions are from the fine-grained recordings between ±2-ms envelope ITD. The ITD functions were classified (Chung et al. 2016) as monotonic (red), trough (green), or other (gray). The bold lines depict the average normalized response across the functions in each class. For the fine-grained recordings shown, the envelope ITD was varied successively between −2 and 2 ms in 25 evenly spaced ITDs or in 167-µs steps, while previously the coarse recording range that extended over a maximum set of ITDs between −8.33 and +28.33 ms with a 1.67-ms ITD spacing was used.

Although plotting neural firing rates, independently of how well these are modulated as a function of ITD, provides one means of characterizing IC neurons’ response to envelope modulations, and will likely influence neurometric measures of ITD discrimination performance (see below), normalizing rate-vs.-ITD functions to the ITD-modulated rate only provides a visually compelling comparison of the effect of different envelope shapes on ITD sensitivity. Figure 6, *A*–*F*, compares normalized spike rates as a function of envelope ITD for all 71 neurons for the same three key envelope shape comparisons as in Fig. 5: PSW vs. SAM, damped vs. ramped, and short duty cycle vs. long duty cycle. The ITD functions were classified according to the method described by Chung et al. (2016) as monotonic (red in Fig. 6), peak, trough (green), or other (gray). Only two examples of a peaking function were found (one for damped and one for ramped stimuli), and so these are not plotted. The bold curves in each panel plot the average normalized response for each class. In each comparison, the envelope shape with the shorter attack and longer pause segment elicits the larger change in spike rate across envelope ITDs. Importantly, this is most evident for ITDs close to zero, i.e., corresponding to frontal spatial locations. The classification of the ITD function was not constant for a given neuron but depended on the stimulus. Compared with envelopes with longer attacks and shorter pauses, envelopes with shorter attacks and longer pause segments elicited a greater proportion of trough and monotonic functions and a lower proportion of unclassified functions.

#### ITD discrimination performance by IC neurons to different envelope shapes.

**Fig. 7.**
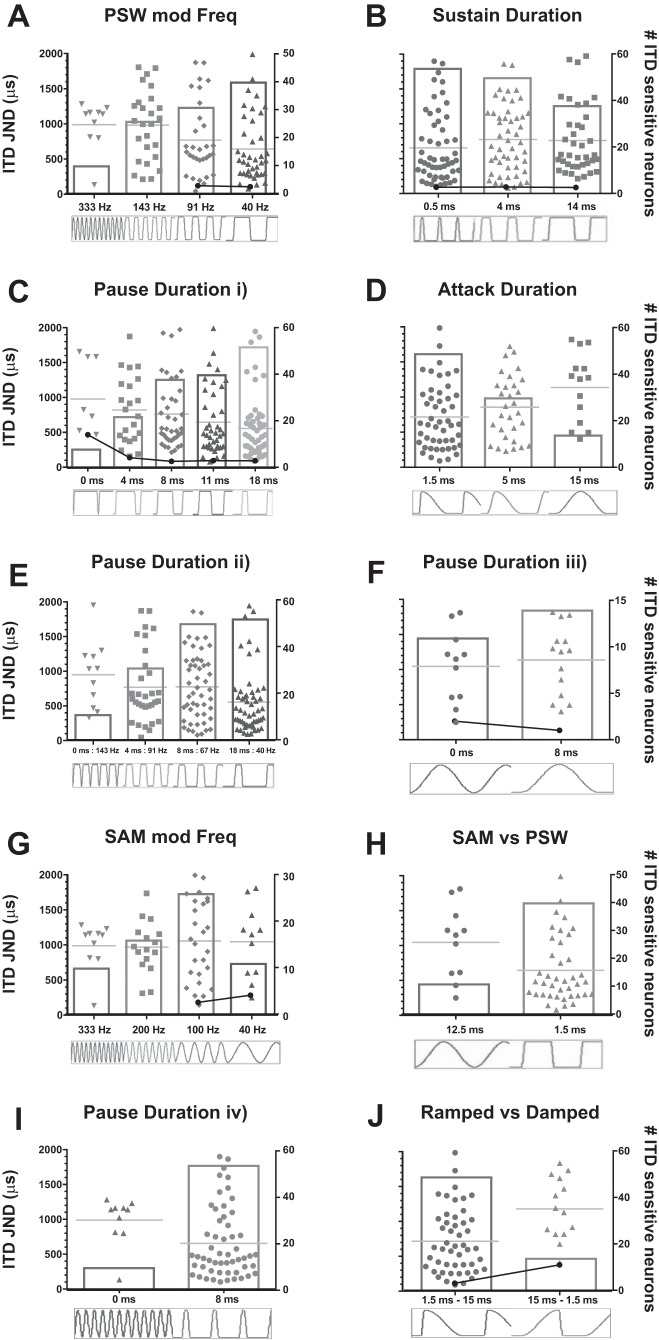
*A–J*: ITD thresholds for each neuron sensitive to the corresponding envelope shape measured within the ±2,000-µs range. The *left* ordinate represents the threshold for ITD discrimination with individual points representing thresholds for individual neurons for D = 1. The gray horizontal line represents the mean threshold of all neurons that reached the threshold criterion. The total number of ITD-sensitive neurons for each envelope shape is plotted on the *right* ordinate and is represented by the vertical bars. Filled circles and solid lines indicate the human psychophysical thresholds measured by Klein-Hennig et al. (2011) for similar envelope parameters (see Table 1). mod, Modulation.

To summarize the influence of the different envelope components on ITD discrimination thresholds, the 18 envelope shapes were assessed with respect to 10 comparison groups. Neural ITD discrimination thresholds were obtained for each neuron and for each envelope shape using the standard separation (D) analysis described in methods. The criterion for accepting a threshold value was D = 1. ITD discrimination thresholds were determined for ITDs that lay within the ±2,000-µs range recorded during the fine-grain measurements and are plotted in Fig. 7, *A*–*J*. In each plot, the *left* ordinate represents the threshold for ITD discrimination (in ms) with the individual points representing thresholds for individual neurons for the particular envelope shape and a value of D = 1. The solid gray horizontal lines represent the mean threshold of all neurons that reached the threshold criterion for a particular envelope shape. In addition to average discrimination thresholds, two other measures of interest are the proportion of neurons in each category exhibiting statistically significant sensitivity to ITDs conveyed by the specific envelope shape and the proportion of these neurons for which the discrimination threshold was <330 μs. To this end, the total number of ITD-sensitive neurons for each envelope shape is plotted with respect to the *right* ordinate and is represented by the vertical bars. Psychoacoustic thresholds from the most closely corresponding conditions tested by Klein-Hennig et al. (2011) are replotted for comparison.

Across all envelope shape comparisons, two main features of the stimulus envelope dominate performance: the duration of the pause and the duration of the attack. Increasing the duration of the pause, independent of the specific envelope shape, results in an increase in both the proportion of neurons exhibiting significant ITD sensitivity and a reduction in the ITD discrimination thresholds. For the 6 envelope shapes assessed with a pause duration of 0 ms (*far left* data sets in Fig. 7, *A*, *C*, *E–I*), the population mean and median thresholds were consistently high, at around 1,000 µs. Across these envelope shapes, an increase in pause duration always resulted in lower average discrimination thresholds and a higher proportion of significantly ITD-sensitive neurons, regardless of changes to other envelope shape segment durations. The substantial influence of pause duration on ITD sensitivity is also demonstrated by the fact that the longest pause duration (18 ms for the 40-Hz PSW plotted in Fig. 7, *C* and *E*, generated the lowest median population threshold (417 µs), the second highest proportion of ITD-sensitive neurons (73%), and the highest proportion of neurons (31%) showing thresholds within the physiological range (±330 µs) (Sterbing et al. 2003).

In general, envelope shapes that elicit the greatest proportion of ITD-sensitive neurons and lowest discrimination thresholds tend to have the widest range of ITD thresholds (due to the small number of relatively ITD-insensitive neurons showing weak ITD sensitivity to these more advantageous envelope shapes), lower threshold for the best-performing neurons, and the greatest numbers of neurons with thresholds contained within the physiological range.

Thresholds of the most sensitive neurons in response to each PSW envelope shape were lowest for the 40- and 91-Hz PSW envelopes at 79 and 46 μs, respectively, increasing to 217 and 134 μs in response to the 143- and 333-Hz PSW. The majority of neurons (76%; 54/71) showed ITD sensitivity to envelope shapes where the sustain duration was relatively short at 0.5 ms (Fig. 7*B*). Increasing the sustain duration to 4 ms and then 14 ms reduced the proportion of ITD-sensitive neurons to 70% (50/71) and 54% (38/71), respectively. Overall, the range of individual ITD thresholds is large for all three envelopes shapes, although the proportion of neurons with low thresholds falls as the sustain duration increases. The most sensitive neurons were found in response to the shortest sustain durations (103 μs at 0.5 ms and 80 μs at 4 ms) and increased in response to the largest sustain duration (214 μs at 14 ms). The importance of the pause duration is also evident in responses to PSW envelopes where both the duty cycle and the modulation rate were kept constant (Fig. 7*C*). Here, the proportion of ITD-sensitive neurons increased from 11% for the shortest (0 ms) pause duration to 31% (22/71) for a 4-ms pause, 38% (54/71) for 8 ms, 56% (40/71) for 11 ms, and 73% (52/71) for the longest (18 ms) pause duration. Furthermore, for the 0-ms pause, no neurons showed threshold lower than the physiological range, whereas nearly one-third of neurons did so for the 18-ms pause, with threshold ITDs of the best-performing neurons falling from 450 to 93 μs between envelope shapes with 0- and 18-ms pause durations.

Reducing the duration of the attack component generally led to improved sensitivity to envelope ITDs. As shown in Fig. 7*D*, reducing the attack duration from 12.5 to 5 ms and then 1.5 ms (*right* to *left*), increases the proportion of ITD-sensitive neurons from 20% to 42% and then 69%, and reduces average thresholds and increases the number of neurons with thresholds within the physiological range. The lowest threshold was 91 μs for the 1.5-ms attack, 229 μs for 5 ms, and 403 μs for 15 ms. The importance of the attack component is most obvious in the comparison of the ramped and damped stimulus shapes (Fig. 7*J*), which are spectrally identical, time-reversed versions of each other. The envelope fast attack (damped) envelope shape generated ITD sensitivity in 69% of neurons, compared with <20% in the time-reversed version, and showed a best-performing neuron with a threshold of 91 µs compared with 407 µs for the ramped stimulus. The relative importance of the attack component, particularly in terms of its primacy over modulation rate per se, can also be inferred by comparing Fig. 7*A* (PSW) and Fig. 7*G* (SAM) but is most evident in Fig. 7*H*, where identical modulation rates (40 Hz) for SAM and PSW are compared directly. The mean ITD discrimination threshold for the envelope shape with the faster attack is approximately half that for the envelope shape with the slower attack. Furthermore, only one neuron showed a threshold for the slow attack component that lay within the physiological range, whereas 18% of neurons in response to the fast attack did so. For most other envelope comparisons, however, it is difficult to dissociate the relative importance of attack and pause components, because these (as well as the sustain and decay components) often covary. Nevertheless, the importance of these components in determining neural performance can be considered with respect to other features often thought important in generating envelope ITD sensitivity, in particular the modulation rate. Across many of the stimulus features it is clear that changing the modulation rate, though modulating ITD sensitivity, is not uniquely important, and indeed, its importance is likely only correlated rather than causal. Figure 7*G*, for example, confirms previous studies indicating preference for rates around 100 Hz. Nevertheless, when a lower rate (40 Hz), for which fewer neurons were sensitive to ITDs conveyed in SAM, was applied in the context of changing the pause duration (Fig. 7*E*), more neurons, and with lower thresholds, showed sensitivity to envelope ITDs at 40 Hz in this condition than the 100-Hz SAM, or with an identical sustain duration or, indeed, a similar modulation rate (91 Hz) to that of the 100-Hz SAM.

**Fig. 8.**
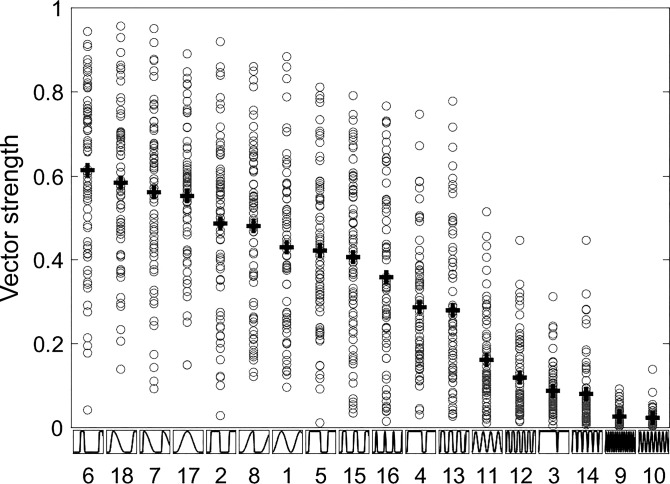
Vector strength values for all 71 neurons (circles), in *left*-to-*right* order of the magnitude of the mean vector strength (crosses). Envelope shapes on the ordinate are numbered according to the condition numbers in Table 1.

The temporal dispersion of action potentials throughout each envelope cycle is likely to be a key factor in determining ITD sensitivity; the more tightly distributed are the action potentials, the more sharply tuned for ITD the neurons are expected to be (Griffin et al. 2005). We assessed the temporal distribution of action potentials evoked within each cycle of the amplitude envelope, generating an analysis of the vector strength (the temporal dispersion of action potentials with respect to the phase of the envelope cycle) for all envelope shapes. Figure 8 plots individual vector strength values for all 71 neurons in *left*-to-*right* order of the magnitude of the mean vector strength (crosses). Clearly, those envelope shapes with the longest pause durations and fastest attacks show the highest vector strengths (*far left*), whereas those with the shortest pause durations (even with fast attacks, such as the 200- and 333-Hz SAM, the poorest performing envelopes) show the lowest (*far right*). Across all neurons and all envelope shapes, there was a small but significant positive correlation of vector strength with firing rate (*r*^2^ = 0.011, *P* < 0.001).

## DISCUSSION

We assessed sensitivity of IC neurons to ITDs conveyed in the envelopes of high-frequency, amplitude-modulated sounds. By comparing neural responses to 18 different modulation shapes and manipulating the attack, decay, pause and sustain segments of the envelope, we have demonstrated the dependence of sensitivity to ITDs conveyed in the amplitude envelope on specific temporal features of the modulated waveform. Specifically, envelope waveforms with sharp attacks and relatively long pauses elicit the best ITD sensitivity in IC neurons. Factors such as modulation rate, often considered important determinants of neural sensitivity to envelope modulations, appear to play a secondary role to the steepness of the attacks and the duration of pauses, as evidenced by the fact, for example, that sinusoidal modulations sometimes elicit little or no ITD sensitivity at rates for which sharper envelope shapes, such as PSW envelopes, show strong tuning for ITD. The neural data are consistent with recent reports of human psychophysical performance where the same features of high-frequency modulated envelopes were assessed.

### 

#### Neural mechanisms of enhanced ITD sensitivity to specific features of the temporal envelope.

From the range of envelope shapes employed in the present study, we can surmise how the output at the level of high-frequency auditory nerve fibers (ANFs), in terms of temporal firing patterns, influences ITD sensitivity. The enhanced ITD sensitivity observed for the PSW, characterized by relatively large changes in spike rate across ITDs (Griffin et al. 2005), is likely influenced by the more precise temporal pattern of action potential generation in high-frequency ANFs in response to those stimulus envelopes with a more rapid onset (Dreyer and Delgutte 2006). As such, the tighter temporal distribution of action potentials (generated monaurally) over the course of the steep envelope attack would lead to a more tightly time-locked firing pattern from each ear converging onto binaural neurons. This explanation is consistent with the explanation posited by Klein-Hennig et al. (2011) for the variation in sensitivity in human listeners to ITDs conveyed in different envelope shapes.

The influence of pause duration on ITD sensitivity observed in the neuron population analysis extends the parameters of the SAM tone, PSW or transposed tones, and was conducted in response to the human psychophysical findings of Klein-Hennig et al. (2011) and Dietz et al. (2013b). The responses to envelope shapes with long pause durations between waveform modulations demonstrate a greater change in spike response rate across different ITDs compared with those envelopes containing short pause durations. This observation is in agreement with the assumption that a pause segment that precedes an attack segment allows for a recovery of sensitivity of neurons within the auditory pathway and that increasing the pause duration from 0 to 18 ms, for example, can result in an improvement in ITD sensitivity. Psychoacoustically, subjects’ thresholds increase markedly as pause duration is increased to 4 ms, and reach almost maximal performance by 8 ms. Our data indicate that although many neurons do not reach peak performance by the 8-ms pause duration, some of the most sensitive neurons do so.

#### Neural sensitivity to modulation rate.

As well as sensitivity to ITDs being greater in response to envelopes with short attack and long pause durations, the neural data show a reduction in ITD sensitivity with increasing modulation rate, consistent with previous electrophysiology studies (Griffin et al. 2005; Yin et al. 1984). A common explanation for the reduced sensitivity to ITD as a function of increasing modulation frequency is the attenuation of spectral components by the limited width of the corresponding peripheral filters. However, this explanation is brought into question by the use of the ramped and damped envelope shapes. These two parameters have equal power spectra but temporally asymmetric envelope shapes and result in ITD JNDs that differ by a factor of ~4 (Klein-Hennig et al. 2011. The response of auditory neurons is known to be greatest at stimulus onset and to be followed by variable levels of adaptation (Smith 1977, 1979), and so the effect of the attack segment is not unexpected. We found shorter (on average, by 10 ms) first-spike latency for the damped compared with the ramped stimulus in line with earlier studies that reported shorter first-spike latencies in response to shorter rise times in the cat primary auditory cortex (Heil 1998). The normalized rate-vs.-ITD function reveals that the damped stimulus elicits the greatest change in spike rate across the physiological range, and the assessment of neuronal ITD JNDs reveals that the ITD JND are generally much lower in response to the damped stimulus, again consistent with the psychophysical findings of Klein-Hennig et al. (2011). From the total population of neurons from which data were obtained, the number of neurons sensitive to envelope ITDs increased when the pause duration segment of the envelope was increased, even for the same modulation rate. The data are also consistent with the enhanced ITD sensitivity generated by transposed tones observed in psychophysical data with human listeners (Bernstein and Trahiotis 2002, 2009). More neurons were sensitive to ITDs within the envelope of high-frequency sounds that had shorter, and therefore steeper, attack segments. Firing rates were more modulated as a function of ITD, and ITD JNDs were consistently lower in response to the PSW than to SAM tones.

#### Accounting for binaural performance.

Perhaps the most striking example of the dependence of ITD tuning on envelope shape comes from the comparison of responses to sounds with identical spectra but temporally reversed envelopes, the so-called damped and ramped stimuli. Despite their identical spectra, ITD tuning was stronger and more frequent (in terms of the number of neurons showing significant modulation of their firing rates with ITD) for the damped stimulus, with its steep attack and slow decay segments, compared with the time-reversed ramped stimulus. Models of binaural processing that take account of total energy, or the interaural correlation of the signals (Bernstein and Trahiotis 1996), cannot account for the differences in ITD sensitivity shown by Klein-Hennig et al. (2011), because both signals are identical in these regards. Even when a stage of peripheral adaptation is added to the cross-correlation model, the sensitivity difference is still underestimated (Klein-Hennig et al. 2011). In a parallel study (Dietz et al. 2016) using modulation cycle histograms of the same data set as in the current study and Hodgkin-Huxley-type model lateral superior olive neurons, we were able to demonstrate that only response onsets of each modulation cycle convey temporal information. Sustained activity, which is rate adapted but in many neurons still well above zero, conveys little, if any, temporal information and is little influenced by ITD. Cross-correlation models do not make this distinction and, consequently, assign too much weight to the sustained component of the envelope.

The precise neural factors underpinning ITD discrimination thresholds remain to be determined. Determining whether behavioral thresholds are pooled across neurons (mean or median threshold), are calculated by some other weighting of neural contributions, or are dominated by neurons with the best discrimination thresholds (the so-called “lower envelope hypothesis”) was beyond the scope of the current study and would have required behavioral measures of ITD sensitivity to be obtained from the guinea pig. Nevertheless, some inferences can be made based on the human psychophysical studies of Klein-Hennig et al. (2011). In particular, the thresholds obtained in those studies for a very similar range of stimulus parameters (i.e., component durations) are consistent with the neural lowest thresholds we observed. For example, thresholds for envelope shapes with a fast attack and decay components were virtually unchanged (~100 μs) as the sustain duration was systematically increased from 0 to 13.1 ms (their Fig. 5). In our data, over a very similar range of sustain durations, the mean of the lowest 10% of thresholds was 165 μs (Fig. 7*B*). Similarly, as pause duration increased from 0 to 17.5 ms in Klein-Hennig et al. (2011), thresholds dropped from ~500 to ~100 μs, a 5-fold reduction reflected in the lowest neural thresholds for almost identical stimuli (Fig. 7*C*). The same authors also reported a 4-fold reduction in human behavioral thresholds for the damped vs. the ramped envelope shapes, from 400 to 100 μs, almost identical to the lowest neural thresholds for these conditions (407 and 91 μs, respectively; Fig. 7*J*). When modulation rates were altered for PSW shapes, however, at least over the range 35–100 Hz (their Fig. 8), there was little change in human psychophysical performance, and this again is reflected in the relatively constant lower bounds of neural performance (and both around 100 μs). This at least comprises circumstantial evidence that behavioral thresholds reflect neural thresholds of the best-performing neurons, rather than the mean or median thresholds.

## GRANTS

This work was funded by a Deafness Research UK Studentship (to D. Greenberg), Medical Research Council Programme Grant 1002267 (to D. McAlpine and T. Marquardt), and the European Union under the Advancing Binaural Cochlear Implant Technology (ABCIT) Grant Agreement No. 304912 (to D. McAlpine, T. Marquardt, and M. Dietz).

## DISCLOSURES

No conflicts of interest, financial or otherwise, are declared by the authors.

## AUTHOR CONTRIBUTIONS

M.D. and D.M. conceived and designed research; D.G. and M.D. performed experiments; D.G., J.J.M.M., and M.D. analyzed data; D.G., J.J.M.M., M.D., T.M., and D.M. interpreted results of experiments; D.G. and J.J.M.M. prepared figures; D.G. and J.J.M.M. drafted manuscript; D.G., J.J.M.M., M.D., T.M., and D.M. edited and revised manuscript; D.G., J.J.M.M., M.D., T.M., and D.M. approved final version of manuscript.
